# Efficacy of ultrasound-guided core-needle biopsy in the diagnosis of hepatic alveolar echinococcosis: a retrospective analysis

**DOI:** 10.1051/parasite/2016019

**Published:** 2016-04-21

**Authors:** Mesut Bulakci, Mehmet Ilhan, Suleyman Bademler, Erdem Yilmaz, Mine Gulluoglu, Adem Bayraktar, Murat Asik, Recep Guloglu

**Affiliations:** 1 Department of Radiology, Istanbul University, Istanbul Faculty of Medicine Istanbul Turkey; 2 Department of General Surgery, Istanbul University, Istanbul Faculty of Medicine Istanbul Turkey; 3 Department of Radiology, Trakya University, Faculty of Medicine Edirne Turkey; 4 Department of Pathology, Istanbul University, Istanbul Faculty of Medicine Istanbul Turkey; 5 Department of Radiology, Medeniyet University, Faculty of Medicine Istanbul Turkey

**Keywords:** Liver, Alveolar echinococcosis, Core-needle biopsy, Ultrasound, Computed Tomography, Magnetic Resonance Imaging

## Abstract

*Background:* This study retrospectively analyzed the clinical data, laboratory results, imaging findings, and histopathological features of 28 patients who underwent ultrasound-guided core-needle biopsy from a hepatic lesion and were diagnosed with alveolar echinococcosis. *Results:* Among 28 patients included in the study, 16 were females and 12 were males. The mean age of the studied population was 53 ± 16 years, and the age range was 18–79 years. The most common presenting symptom was abdominal pain, which was observed in 14 patients. A total of 36 lesions were detected in the patients’ livers, out of which 7 had a cystic appearance. Hepatic vascular involvement, bile duct involvement, and other organ involvement were depicted in 14, 5, and 7 patients, respectively. The average number of cores taken from the lesions was 2.7, ranging between 2 and 5. In histopathological evaluation, PAS+ parasitic membrane structures were visualized on a necrotic background in all cases. Regarding seven patients, who were operated, the pathological findings of preoperative percutaneous biopsies were in perfect agreement with the pathological examinations after surgical resections. None of the patients developed major complications after biopsy. *Conclusion:* Ultrasound-guided core-needle biopsy is a minimally invasive, reliable, and effective diagnostic tool for the definitive diagnosis of hepatic alveolar echinococcosis.

## Abstract (fr)

*Contexte*: Cette étude a analysé rétrospectivement les données cliniques, les résultats de laboratoire, les résultats d’imagerie, et les caractéristiques histopathologiques de 28 patients qui ont subi une biopsie guidée par échographie d’une lésion hépatique et ont été diagnostiqués avec échinococcose alvéolaire.

*Résultats*: Parmi les 28 patients inclus dans l’étude, 16 étaient des femmes et 12 étaient des hommes. L’âge moyen de la population étudiée était de 53 ± 16 ans, et la tranche d’âge était 18-79 ans. Le symptôme le plus fréquent était la douleur abdominale, qui a été observée chez 14 patients. Un total de 36 lésions a été détecté dans le foie des patients, dont sept avaient une apparence kystique. L’atteinte hépatique vasculaire, l’implication des voies biliaires, et l’atteinte d’autres organes ont été trouvées chez respectivement 14, 5 et 7 patients. Le nombre moyen de carottes prélevées à partir des lésions était de 2.7 et compris entre 2 et 5. Dans l’évaluation histopathologique, des structures membranaires parasitaires PAS+ ont été visualisées sur un fond nécrotique dans tous les cas. En ce qui concerne 7 patients qui ont été opérés, les résultats pathologiques des biopsies percutanées préopératoires étaient en parfait accord avec les examens pathologiques après résections chirurgicales. Aucun des patients n’a développé de complications majeures après la biopsie.

*Conclusion*: La biopsie guidée par échographie est un outil de diagnostic minimalement invasif, fiable et efficace pour le diagnostic définitif de l’échinococcose alvéolaire hépatique.

## Introduction

Alveolar echinococcosis (AE) is a rare zoonotic infection that affects humans as coincidental intermediate hosts. The infection is contracted by humans via ingesting adult *Echinococcus multilocularis* eggs. AE is a progressive disease that can result in death unless treated. Its primary site of involvement is the liver, from where it may spread to nearby organs by direct invasion, or distant organs by metastasis. The metacestode stage of the parasite leads to the formation of slowly growing, infiltrative, tumor-like mass lesions in affected organs. As a result of these characteristics, AE lesions may easily be confused with malignant lesions [13].

It is vital that affected patients are diagnosed early in the course and treated accordingly. The disease is diagnosed with the help of clinical signs along with epidemiological data, typical radiological signs, and serological tests (probable AE) [6, 15, 16]. To confirm the diagnosis of the disease (proven AE), however, histopathological examination should be compatible with AE or the organism’s nucleic acid should be detected in a clinical sample obtained from a patient [6].

AE is endemic in some rural areas of Turkey; as such, many cases have been admitted to our hospital for the diagnosis and management of AE. Ultrasound-guided core-needle biopsy has been successfully performed in suspected AE cases at our department for many years. However, there is an insufficient amount of information about the role of this procedure in the diagnosis of AE. Herein, we share our experience in ultrasound-guided core-needle biopsy for the diagnosis of AE and present the associated retrospective analysis comprising the clinical, laboratory, and imaging findings of the patients.

## Patients and methods

### Ethics statement

This study protocol was approved by our hospital Ethics Committee (2015/1286). Each patient received details of ultrasound-guided core-needle liver biopsy and provided written informed consent for the procedure.

### Patient selection

This study enrolled patients who presented to our hospital with various complaints and were diagnosed with AE by core-needle biopsy taken from a hepatic lesion detected by various investigations between January 2008 and July 2015. Presenting complaints, pre-biopsy imaging findings, and laboratory results were retrospectively analyzed using our hospital’s patient archive system.

### Biopsy procedure

Biopsy procedures of all patients were performed at the radiology department, under adequate aseptic conditions and local anesthesia. Local anesthesia was done under sonographic guidance at the expected pathway of the needle biopsy with a 22-gauge needle in a 10-mL syringe filled with 2% prilocaine HCl. Bleeding parameters (performed within 2 weeks before the procedure) and hemograms were checked in all patients prior to the procedure. The required values of the parameters before percutaneous biopsy were as follows: Platelet count > 70,000/mL [150,000–450,000/mL], international normalized ratio (INR) < 1.5 [0.85–1.25], and activated partial thromboplastin time (aPTT) < 40 s. Patients, who were using anticoagulants, stopped their medications one week prior to the procedure.

All lesion biopsies were performed under real-time ultrasound guidance (Xario, Toshiba Medical Systems Corporation, Tochigi, Japan) using a low-frequency (3.5 MHz) convex probe with a multiple-pass technique. At the start of the biopsy procedure, the relation of a lesion with major vascular structures and intralesional vascularity was evaluated by color Doppler examination to determine the tract of the needle. The content and organization of a lesion were sonographically evaluated (in combination with computed tomography [CT] and/or magnetic resonance imaging [MRI]) to determine the best sampling site. The procedure was performed as a free-hand technique using a 16-cm long, 18-gauge (18G) cutting needle, which can take a sample having a length of approximately 2 cm. In order for the needle to easily pass through skin, a small incision was made with a No: 11 blade. The patient position and the approach to be applied depended on the localization of a lesion and operator’s preference. An intercostal or subcostal approach in left lateral decubitus position was mostly preferred for lesions located in the right lobe, while a subcostal or substernal approach in normal decubitus position was preferred in lesions located in the left lobe.

After visually confirming that an adequate amount of sample was taken, the procedure was ended, and the patients were followed up clinically for 2 h after the procedure before discharge. Potential complications of the biopsy procedure were classified as major and minor. The complications that would require urgent intervention and that would result in death, unless treated, were classified as major, while those that would only require monitoring or conservative treatment were classified as minor. Potential complications and presence of free fluid in the abdominal cavity were assessed by sonographic examination at the end of the routine monitoring period.

### Pathologic examination

As the standard laboratory procedure, the core-needle biopsy specimens were processed and embedded in paraffin blocks. Microscopic examinations were performed in hematoxylin and eosin (H&E) and periodic acid-Schiff (PAS) stained 3-μm thick sections by a single pathologist who is experienced in liver pathology (MG).

## Results

### Patients

During the study period, a total of 28 patients (12 males and 16 females) were enrolled. The mean age of the study population was 53 ± 16 years, and the age range was 18–79 years. The diagnosis of AE was coincidental in 18 patients (i.e., the diagnosis emerged after the biopsy), while in the rest of the cases, AE was considered in the list of differential diagnoses. None of the biopsies caused major complications, such as bleeding that required blood transfusion or intervention, or anaphylactic reactions. Short-lasting mild-to-moderate right upper quadrant and right shoulder pain were observed in six patients after the procedure. Furthermore, parasitic tissue seeding along the biopsy tract was not detected after the biopsies for a follow-up period ranging from 2 to 90 months. The median times of follow-up for operated patients, unoperated patients, and all cases are 27, 53, and 37 months, respectively. As a result of clinical and radiological studies, 21 patients were deemed inoperable and treated with albendazole. While lifelong treatment with albendazole is administered in unresectable patients and patients with R2 resection, patients with R0 and R1 resection are postoperatively treated for 2 years. The treatment with medication is administered for 3 weeks and then discontinued for 1 week. A follow-up MRI of the abdomen is performed for patients with R0 and R1 resection every six months during the first two postoperative years and then once a year. Unenhanced CT scan of the thorax is also performed annually in the postoperative period. Unresectable patients and patients with R2 resection are followed up with MRI of the abdomen and magnetic resonance cholangiopancreatography (MRCP) once a year unless there is a clinical or biochemical problem. A biliary drainage procedure was successfully performed for bile duct decompression in three patients. Cavitary lesions of four patients were evacuated by percutaneous drainage without any complication under sonographic guidance. The clinical characteristics, imaging results, and treatment regimens of our patients are summarized in Table 1.

**Table 1.** Clinical and imaging characteristics of the patients.

Case	Sex	Age	Primary symptoms	Hepatic lesion location/size	Vascular involvement	Biliary involvement	Organ involvement	PNM	Treatment
1	M	65	Fever, abdominal pain	R-15 × 12 cm	RHV, RPV	−	Lungs, R-Adrenal gland, Pancreas	P2N1M1	PD, CT
2	M	60	Asymptomatic	R-14 × 8 cm	−	−	−	P1N0M0	?
3	M	38	Abdominal pain	R-19 × 17 cm	RHV, MHV, RPV	−	Lymph nodes	P3N1M0	PD, CT
4	F	39	Anorexia, nausea	R-16 × 8 cm	−	−	−	P1N0M0	R-Hepatectomy + CT
5	F	43	Asymptomatic	R-4 × 3 cm	−	−	−	P1N0M0	?
6	F	64	Anorexia	R-11 × 7 cm + 8 × 5 cm	−	−	−	P1N0M0	PD, CT
7	F	79	Abdominal pain	R & L-16 × 14 cm	PV	−	−	P4N0M0	CT
8	F	51	Back pain	L-10 × 8 cm	PV, RPV, HA	+	−	P4N0M0	CT, Tx
9	F	61	Abdominal pain, jaundice	L-9 × 7 cm	LHV, LPV	+	−	P2N0M0	PBD, CT
10	M	36	Abdominal pain	R-17 × 15 cm, L-6 × 6 cm	RHV, MHV, RPV	−	−	P3N0M0	PD, CT
11	F	34	Abdominal pain	R-10 × 8 cm + 10 × 7 cm	−	−	−	P1N0M0	CT
12	F	56	Abdominal pain	R-11 × 8 cm + 9 × 7 cm	−	−	−	P1N0M0	CT
13	F	67	Abdominal pain, malaise	R-15 × 11 cm	RHV, RPVP	−	Lymph nodes	P2N1M0	CT
14	M	27	Abdominal pain	R-8 × 6 cm	−	−	−	P1N0M0	Mass Excision + CT
15	F	23	Headache, nausea, vomiting	R-15 × 14 cm	RHV, RPV, IVC	−	Brain, Lungs, R-Adrenal gland	P4N1M1	CT
16	F	68	Weakness, anorexia	R & L-8 × 7 cm	−	+	−	P2N0M0	PBD, CT
17	F	51	Abdominal pain, dispnea	R-19 × 17 cm, L-5 × 3 cm	RHV,RPV	−	−	P1N0M0	CT
18	M	61	Malaise	R-6 × 3 cm, L-4 × 3 cm	−	−	Lymph nodes	P1N1M0	?
19	M	67	Asymptomatic	R-13 × 7 cm	−	−	−	P1N0M0	R-Hepatectomy + CT
20	M	68	Multiple myeloma	R-16 × 14 cm + 5 × 4 cm	RHV, RPV	−	R-Adrenal gland	P2N1M0	CT
21	F	55	Jaundice, itching	R & L-17 × 15 cm	MHV	+	−	P2N0M0	PBD, CT
22	M	60	Malaise	L-10 × 7 cm	−	−	−	P1N0M0	L-Hepatectomy + CT
23	M	18	Abdominal pain	R & L-12 × 6 cm	−	−	−	P1N0M0	CT
24	F	50	Abdominal pain	R-11 × 6 cm	RHV, RPVP, IVC	−	R-Adrenal gland	P4N1M0	R-Hepatectomy + CT
25	F	75	Abdominal pain	R-20 × 10 cm	RPV	−	−	P3N0M0	CT
26	M	78	Gastric cancer	R-8 × 8 cm	−	−	−	P1N0M0	CT
27	F	43	Abdominal pain, malaise	R & L-17 × 11 cm	LHV, LPV	+	−	P2N0M0	CT, Tx candidate
28	M	59	Weakness, malaise	L-9 × 6 cm + 6 × 5 cm	−	−	−	P1N0M0	L-Lobectomy + CT

RHV: Right Hepatic Vein, MHV: Middle Hepatic Vein, LHV: Left Hepatic Vein, PV: Portal Vein, RPV: Right Portal Vein, LPV: Left Portal Vein, RPVP: Right Portal Vein Posterior branch, IVC: Inferior Vena Cava, HA: Hepatic Artery, PD: Percutaneous Drainage, PBD: Percutaneous Biliary Drainage, CT: Chemotherapy, Tx: Liver transplantation.

### Clinical and laboratory findings

Twenty-four of our patients were clinically symptomatic. In the laboratory analyses of 25 patients, at least one biochemical parameter was above the normal values. Abdominal pain discomfort was the most common complaint affecting 14 patients. Three patients were asymptomatic. Two patients had jaundice and itching due to bile duct involvement, while a patient with brain metastases had headache, nausea, and vomiting. Two other patients were noted to have mild weight loss. Serum bilirubin levels (total and direct bilirubin), transaminase levels (aspartate aminotransferase [AST] and alanine aminotransferase [ALT]), and cholestatic enzymes (Gamma-glutamyl transpeptidase [GGT] and alkaline phosphatase [ALP]) were, respectively, elevated in 4 patients, 4 patients, and 12 patients. Only one patient, who had been operated for gastric cancer, had an elevated cancer antigen 19-9 (CA 19-9) level (73 u/mL). One patient had a minimally elevated carcinoembryonic antigen (CEA) level (6.19 ng/mL), and another patient had an elevated alpha-fetoprotein (AFP) level (94.55 ng/mL). All other patients had normal levels of AFP, CEA, and CA 19-9. The laboratory results on admission are listed in Table 2.

**Table 2.** Baseline data of the study population.

Patient	28		
Gender: Male/Female	12/16		
Age: mean (range)	53 (18–79)		
Laboratory results	Mean (range)	Normal range of value	Number of patients with value outside the range
AST	35 (12–290)	5–42 U/L	1
ALT	30 (8–192)	10–40 U/L	4
ALP	266 (30–1895)	40–129 U/L	12
GGT	105 (20–530)	5–85 U/L	10
LDH	365 (153–658)	135–250 U/L	21
Total Bilirubin	1.06 (0.2–14)	0.2–1 mg/dL	4
Direct Bilirubin	0.74 (0.01–13.39)	0–0.3 mg/dL	4
Total Protein	7.64 (6.3–8.7)	6.4–8.3 g/dL	5
AFP	7.78 (0.9–94.55)	<13.6 ng/mL	1
CEA	2.06 (0.23–6.19)	<5 ng/mL	1
CA 19-9	18.6 (2.3–73)	0–34 U/mL	1

AST: Aspartate aminotransferase, ALT: Alanine aminotransferase, ALP: Alkaline phosphatase, GGT: Gamma-glutamyl transpeptidase, LDH: Lactate dehydrogenase, AFP: Alpha-fetoprotein, CEA: Carcinoembryonic antigen, CA 19-9: Cancer antigen 19-9.

### Imaging features

**Figure 1.**
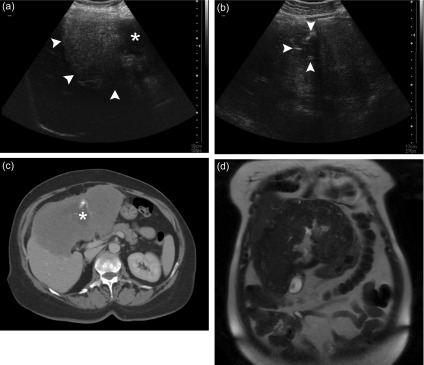
A 43-year-old female patient presented with abdominal pain and malaise. (a) Abdominal ultrasound examination reveals a hyperechoic, heterogeneous solid mass lesion that fills part of the right lobe and the entirety of the left lobe of the liver. Its borders can be clearly discerned from the adjacent normal hepatic parenchyma (*arrowheads*). The necrotic cavity in the central zone of the lesion appears as a hypoechoic area with irregular contours (*asterisk*). (b) Calcifications around the central necrotic zone can be discerned as hyperechogenic foci (*arrowheads*). (c) An axial contrast-enhanced CT image demonstrates the hypovascular solid mass lesion more clearly. The necrotic cavity in the left lobe (*asterisk*) and the tiny calcifications around it are visible. Additionally, note that the lesion causes a retraction in hepatic contours. (d) A coronal T2W MRI image shows a heterogeneous mass lesion that is mildly hypointense relative to liver parenchyma. While the necrotic cavity in the center of the lesion appears of high signal intensity, the calcifications are not easily discernible.

**Figure 2.**
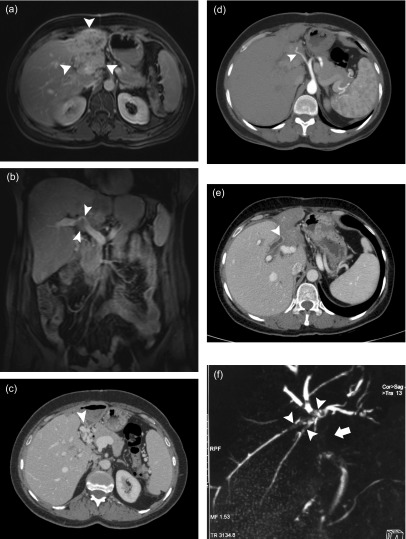
A 51-year-old female patient presented with back pain. (a) Abdominal MRI with contrast demonstrates a mass lesion (*arrowheads*) with indistinct borders, which completely fills the left lobe of the liver with concurrent atrophy and shows diffuse heterogeneous enhancement. (b) Postcontrast coronal MRI image demonstrates portal vein invasion and narrowing (*arrowheads*). (c) Many collateral veins (*arrowhead*) that developed at the hilus secondary to portal vein invasion are demonstrated by portal phase CT examination. (d) An arterial phase axial CT image reveals the hepatic artery wrapped by the mass (*arrowhead*). (e) Contrast-enhanced abdominal CT examination shows that the contrast-enhanced, thickened common bile duct is interrupted within the lesion (*arrowhead*). (f) Filling defects due to the bile ducts invaded by the mass (*arrowheads*) and the interruption of the common bile duct (*arrow*) are more clearly visualized by MRCP images. Cholangiocarcinoma was considered in the differential diagnosis; however, the core biopsy result was consistent with AE.

**Figure 3.**
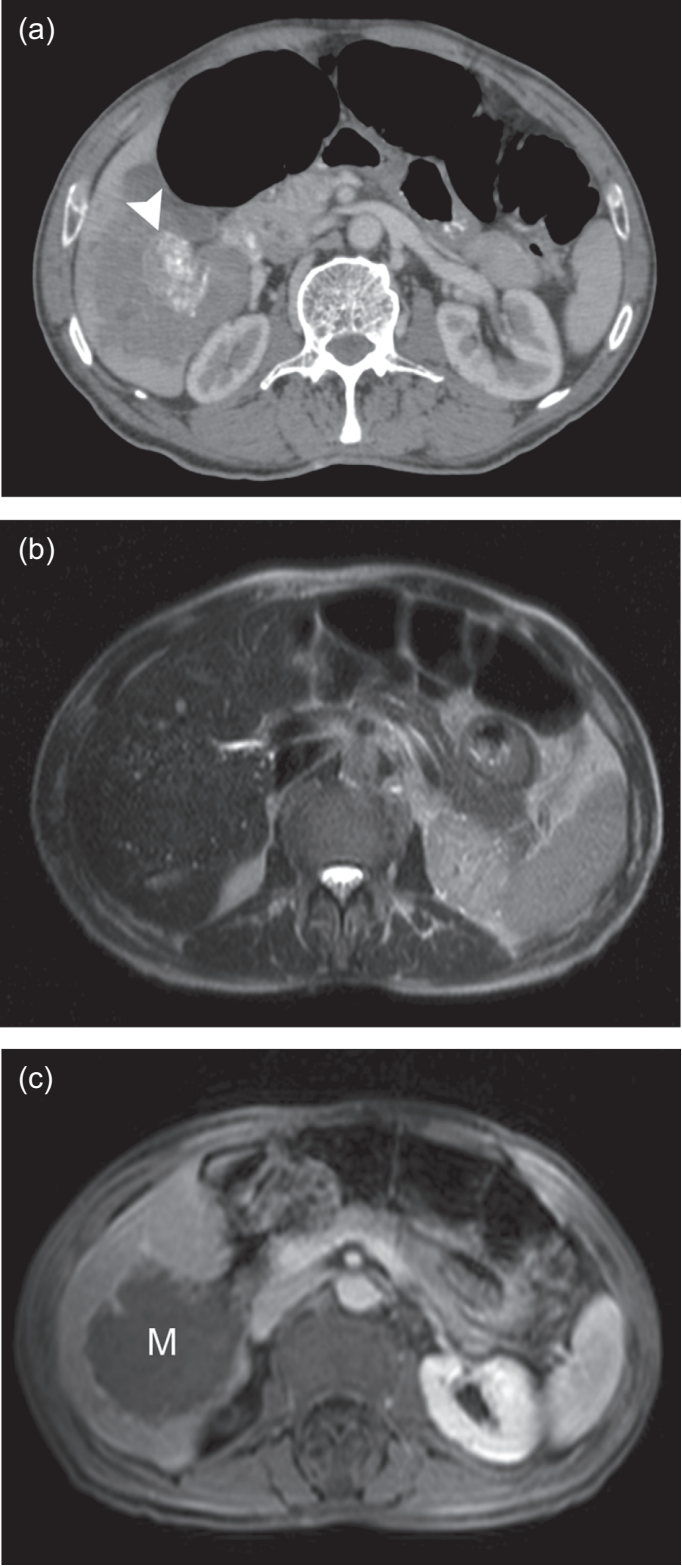
A 78-year-old male patient previously operated for gastric cancer was found to have a mass in the right lobe of the liver. (a) Axial contrast-enhanced CT examination shows an irregularly bordered, hypovascular, heterogeneous mass lesion in the right lobe. Focal calcific areas (*arrowhead*) in the center of the lesion can be clearly discerned. (b) On an axial T2W MRI image, the mass is not clearly discernible from the normal liver parenchyma. However, many hyperintense, small cysts can be seen at the peripheral zones of the lesion. (c) A postcontrast axial MRI image demonstrates that the mass (*M*) did not show prominent contrast uptake with the exception of a weak, peripheral contrast uptake. With an elevated CA 19-9 level, the patient underwent core biopsy to exclude a malignancy, and the result indicated the diagnosis of AE.

Twenty-eight patients underwent percutaneous biopsy, a total of 36 lesions were detected. There was a single hepatic lesion in 20 patients while two lesions were found in 8 patients. All (100%) of these lesions could be visualized sonographically at the time of biopsy. At least one cross-sectional examination existed in all patients; 23 of them had a CT examination and 20 patients had an MRI examination. Twenty-three of the lesions were located in the right lobe, eight in the left lobe, and five in both lobes. Calcifications were detected by CT in 19 patients (82%), whereas calcifications were detected by ultrasonography (US) in only 17 patients (60%). Necrotic cavity formation was detected by MRI in 12 patients (60%), by CT in 11 patients (48%), and by US in 14 patients (50%). All lesions appeared hypodense in contrast-enhanced CT; however, no intralesional contrast uptake was detected. In sonography, 16 lesions appeared heterogeneous hyperechoic, 13 lesions appeared heterogeneous isoechoic, and 7 cystic lesions appeared hypoechoic. While the borders of 24 lesions could not be clearly discerned, 12 lesions had clearly discernible borders. On T1W MRI series, 16% of the lesions were isointense and 84% of the lesions were hypointense, while, on T2W MRI series, 42% of the lesions were isointense, 33% were mildly hypointense, and 25% were heterogeneous hyperintense. Except for a mild-moderate peripheral contrast uptake, no intralesional contrast uptake was present on postcontrast images, with an exception of one case. The distribution of the 24 lesions found in the 20 patients who underwent MRI based on Kodama’s classification was as follows: type 2 (3 lesions), type 3 (8 lesions), type 4 (6 lesions), and type 5 (7 lesions). We did not observe any lesion of type 1 in our series. Retraction and irregularity of the liver capsule were observed in ten patients. Right-lobe atrophy was present in two patients and left-lobe atrophy was present in five patients. Twelve patients had hepatomegaly, and one patient had splenomegaly. Bile duct dilatation could be demonstrated in each of the three examinations in five patients. Eleven patients were shown to have hepatic vein involvement and 13 patients had portal vein involvement. In one patient, the mass was encircling the hepatic artery and its left branch. Four patients had surrenal gland involvement (all right-sided), and three patients had intraabdominal lymph node involvement. Two patients had lung metastasis, one had brain metastasis, and one had pancreatic involvement. Demonstrative US, CT, and MRI images of hepatic AE obtained from three different patients are shown in Figures 1–3.

### Pathological features

**Figure 4.**
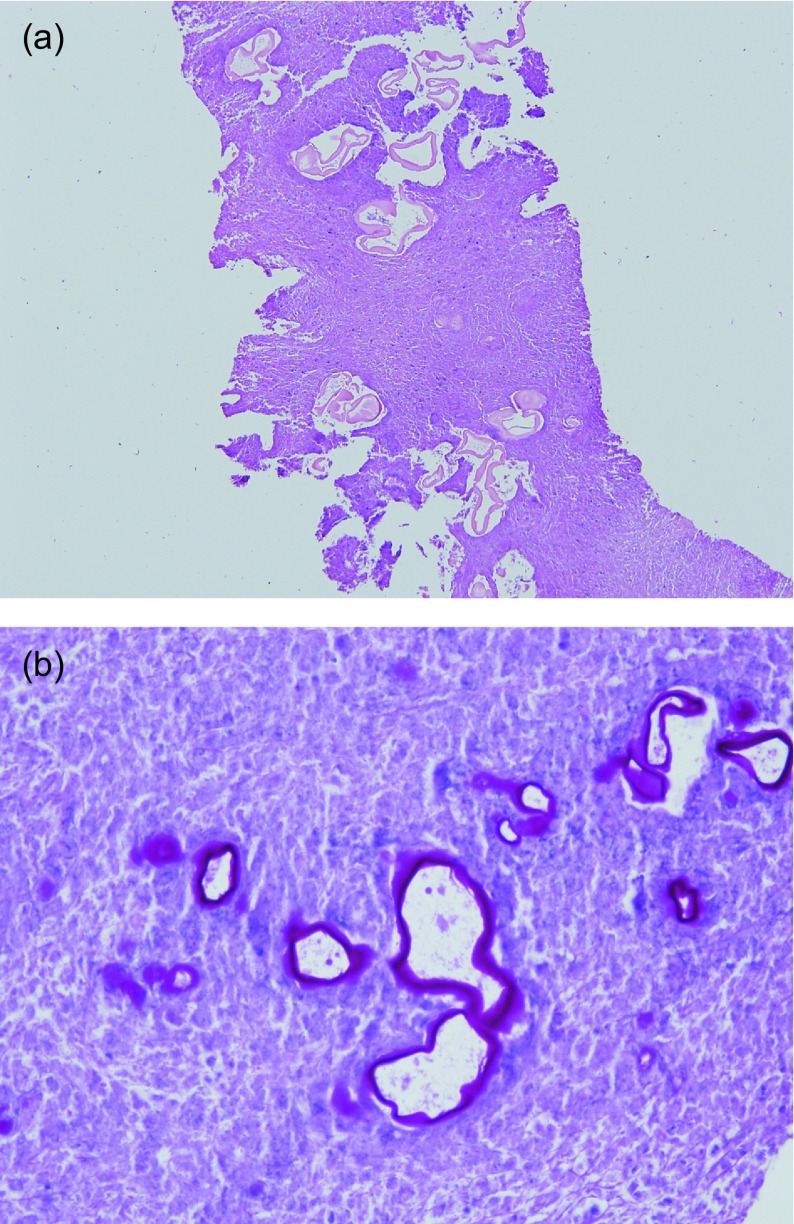
(a) Pale eosinophilic laminated membrane particles in the necrotic background (H&E, original magnification 200×). (b) The membrane particles were highlighted by PAS histochemical stain (PAS, original magnification 400×).

The mean number of cores was 2.7 (range: 2–5). Microscopic evaluation revealed that the samples consisted of necrotic debris containing PAS (+) small laminated membrane particles, which is a characteristic of alveolar-type echinococcosis (Fig. 4). Some of the biopsies contained areas of fibrosis, chronic inflammation, and granulomatous reaction adjacent to necrotic areas. Calcifications were present in only two patients’ biopsy preparations. Biopsy materials were adequate for definitive diagnosis in all patients, and there was no need to perform a repeat biopsy in any of the patients. In seven patients, who were operated, the pathological examination of surgically and percutaneously obtained samples was perfectly concordant.

## Discussion

Alveolar Echinococcosis continues to be a serious disease that poses both diagnostic and therapeutic challenges to most clinicians. The majority of affected patients are chronic carriers of the disease who require uninterrupted medical treatment and follow-up. Unfortunately, treatment options are limited due to delayed diagnosis in most cases [16]. Although patients undergoing discontinuous treatment were considered in this study, uninterrupted treatment is currently recommended (Strength of recommendation: B Quality of Evidence: III) [6]. The disease has an asymptomatic initial phase that lasts for 5–15 years on average. At this stage, spontaneous cure can take place, or the disease passes on to the progressive phase, in which patients become symptomatic depending on the organ system(s) involved. In the advanced stage, severe hepatic dysfunction develops, which is commonly accompanied by portal hypertension [13]. Affected patients may present symptoms of abdominal pain, cholestatic jaundice, weight loss, fatigue, and less commonly, fever and anemia [13, 20]. On the other hand, more than a third of patients are coincidentally diagnosed [15].

Plain radiograms, ultrasonography, CT, MRI, positron emission tomography-computed tomography (PET-CT), and contrast-enhanced ultrasonography are used for imaging AE cases. Ultrasonography, by virtue of its low cost and rapid diagnostic abilities, is preferred for screening large populations in endemic regions. It has been reported that this modality is a feasible and effective method in diagnosing AE cases [3]. Sonography also guides interventional procedures used for the diagnosis and palliative treatment of these cases. In ultrasonography, AE lesions typically (70% of cases) appear as large heterogeneous masses with irregular borders, in the center of which hypoechoic cystic-necrotic areas and calcific foci are visualized. Less commonly, AE lesions may also appear as multiple, hemangioma-like hyperechogenic solid lesions (hailstorm pattern), small calcified areas, or cystic lesions containing massive necrosis. Lesion borders are not clearly discerned, and they usually appear hyperechogenic [3, 5, 10]. An AE lesion may be confused with a cystadenoma-cystadenocarcinoma or hydatid cyst when its appearance is largely cystic [5]. This appearance corresponds to the type 5 pattern in the classification developed by Kodama et al. [17]. In our study group, we did not encounter the hailstorm pattern or patterns of small calcific foci. Twenty-three patients had lesions with a solid appearance, while 5 patients had cystic lesions with dense content. When compared with the series reported by Kodama et al. [17] and Azizi et al. [1], lesions of types 4 and 5 that are thought to indicate an advanced stage in the course of the disease were noticeably more frequent in our series. When compared with the series reported by Becce et al. [4], there were more lesions of type 4 and fewer lesions of type 2. In one of our cases, the CA 19-9 level was considerably elevated in the sample obtained from the cyst cavity and, thus, cystadenocarcinoma was considered in the differential diagnosis; however, a core biopsy from the cyst wall confirmed the AE diagnosis. Hydatid cyst disease was considered in the differential diagnosis in the other four patients. In a recently published study, it was reported that particularly pseudo-cystic AE lesions (type 5 lesions in the Kodama classification) could be misdiagnosed as cystic echinococcosis (CE) and, thus, the patients could undergo treatments that cause them harm [24]. We agree with this opinion of the authors. In other words, utmost caution is required when examining mostly cystic lesions, and when needed, histopathological analysis and supporting diagnostic tools, such as immunohistochemical staining or PCR, should be used.

Computed tomography (CT) and magnetic resonance imaging (MRI) are necessary for staging and preoperative assessment of AE lesions. In both modalities, the number, anatomic site(s), morphological properties, and spread of AE lesions can be more thoroughly delineated. Involvement of vascular structures and bile ducts can also be evaluated. While CT is the most valuable modality particularly for showing characteristic calcifications (in 90% of cases), MRI (particularly T2W series) is more effective in showing cystic structures of the parasite. Lesions appear hypodense on CT and hypointense in T1W MR images [10]. In T2W MRI series, on the other hand, lesions may have an iso-, hypo-, or hyperintense appearance [10, 17]. The lack of contrast uptake, apart from mild contrast enhancement in the lesion periphery, is an important diagnostic feature in contrast-enhanced CT and MRI examinations [10, 15]. In contrast with these previously reported data, we detected one lesion with an aggressive appearance pattern showing diffuse, moderate, and heterogeneous contrast uptake in one of our patients. A core biopsy, which was taken because of a strong suspicion of a malignancy in the differential diagnosis, then revealed findings that were consistent with AE. When compared with other lesions, parasitic tissues and necrotic areas of this lesion were sparser in the microscopic examination. On the other hand, the periparasitic inflammatory response was more prominent. These pathological findings can clarify the aforementioned radiological appearances.

In cases when the immune system is suppressed, AE may be present in different clinical forms, and AE lesions may exhibit atypical imaging properties [9, 18]. Furthermore, it is reported that serological tests could yield false negative results in such cases [9]. In these cases, histopathological confirmation supported by immunohistochemical staining and PCR becomes particularly important [9]. Imaging-guided core biopsy and fine-needle aspiration biopsy (FNAB) have been used for diagnosing hepatic lesions with great success for many years. Needle-core biopsy is the gold standard for histopathological diagnosis of hepatic masses [14]. Both techniques have their own advantages and disadvantages. The main advantages of core biopsy include the preservation of tissue structure during biopsy sampling, general pathologists being more familiar with the lesion’s histopathology, and additional advanced examinations being simpler to perform in the biopsy specimen [14]. Histopathological examination is used for the confirmation of hepatic AE lesions. This is achieved by the examination of biopsy material obtained percutaneously – via FNAB or core biopsy – or tissue samples excised surgically. Ultrasound-guided core-needle biopsy is the main preferred technique for establishing the pathological diagnosis of solid hepatic lesions in our hospital. Despite being successful, it is crucial in this method to take biopsy samples in sufficient amounts and from appropriate locations. Furthermore, a pathologist should be familiar with the lesion’s histopathological properties. One case report showed that inflammatory pseudotumor was considered in the differential diagnosis of an AE case, and ultrasound-guided FNAB and core biopsy (taken twice) failed to make the diagnosis [19]. With respect to our findings, in pathological evaluation, the visualization of PAS+ membrane structures of parasitic origin in tissue sections is the most important diagnostic criterion. Hence, to make an accurate and definitive diagnosis, biopsy material should contain parasitic tissues. Accordingly, the reason for not being able to make a diagnosis with percutaneous biopsy in the above-mentioned case report of Madhusudhan et al. [19] might have been the fact that the material obtained by sampling did not contain parasitic membrane tissues but only perilesional fibroinflammatory tissues.

Based on the aforementioned analysis, the critical aspect is the region of the lesion to be sampled to make an accurate diagnosis of AE lesions that have already reached a large size at the time of admission. As hepatic AE lesions grow in size, widespread areas of necrosis develop due to insufficient vascular supply [15]. Furthermore, the intermediate host responds to the parasitic invasion by the formation of a periparasitic granuloma that is characterized by intense chronic inflammatory infiltration containing foreign body giant cells surrounding metacestodes, and accompanying diffuse fibrosis [25]. Findings accompanying these metacestodal structures are nonspecific, with none of them alone being sufficient to make AE diagnosis. MRI is the best imaging modality that reveals different components and the vesicular structures of the parasite [5]. On the other hand, it is now a widely accepted view that areas of peripheral enhancement in contrast-enhanced CT and MRI and increased peripheral FDG uptake in PET-CT imaging originate from an inflammatory reaction rather than parasitic tissues [8, 15]. Hence, in contrast to neoplastic hepatic masses, sampling of these areas showing uptake in PET-CT may lead to a false negative pathology result in AE lesions, while sampling from metabolically active zones is usually aimed at malignant lesions. On the other hand, Azizi et al. [1] advocated that microcystic parasitic structures visualized in T2W MRI series are correlated with metabolically active disease. This corresponds to types 1–3 in the Kodama classification. We observed in the pathological examination of our cases, that the most important feature accompanying parasitic structures was intense necrosis, which was also reported as the most prominent pathological feature in the previously published FNAB study [11]. Based on these data, one may conclude that sampling from central or necrotic zones of a lesion rather than its peripheral zones may increase the likelihood of making an accurate diagnosis. Parasitic structures cannot be clearly distinguished by sonography. However, it has been reported that hypoechoic areas within a lesion contain active parasitic tissues [5]. We suggest that taking a few samples from different zones of a lesion and from different depths will increase the likelihood of diagnosis. In addition, a pre-procedural MRI may guide accurate sampling of a lesion. We also think that sampling from several localizations of thick cyst wall will suffice for the diagnosis in core biopsies of cystic lesions categorized as type 5 by the Kodama classification.

In regions where AE and CE are co-endemic, such as Turkey, it is critical to be aware of the histopathological differences between these diseases with regard to liver involvement. Echinococcus metacestodes are surrounded by a carbohydrate-rich acellular layer, which is referred to as the laminated layer, inside the host tissue where they reside [12]. This structure, which is formed by the cells in the germinal layer just beneath it, is a hallmark in the pathological diagnosis [2, 12]. The germinal layer develops protoscoleces by budding toward the inside. These structures exist in all Echinococcus larvae although variations are observed [12]. In the conventional pathological examination, it is vital to observe a strong PAS-positive laminated layer due to its high amount of polysaccharide content. This is because, in humans, protoscoleces and hooklets are rarely seen [2]. The histopathological differentiation of AE and CE is mainly based on the different growth patterns of their metacestodes [2]. By budding toward the outside into the host tissue, *E. multilocularis* metacestodes generate complex lesions that consist of multi-cystic, tubular structures resembling a labyrinth [2, 12]. These formations are surrounded by heavy inflammatory cells and necrotic areas that do not show certain boundaries [2]. *E. granulosus* metacestodes, however, generate a larger solitary cyst by budding toward the inside, which has a thicker laminated layer (10–12 μm in AE, up to 5 mm in CE) showing concentric growth. This cyst is filled with a high-pressure fluid and is surrounded by a fibrotic capsule (adventitial layer) that is formed by the host tissue with clear boundaries. Perilesional inflammation and necrosis are not distinctive in contrast to AE [2, 12].

Despite all the above-mentioned distinguishing features, diagnostic difficulties arise in these two pathologies. Barth et al. [2] have shown in their studies including 96 patients and paraffin blocks obtained from various organs that there were diagnostic difficulties in standard pathological examination of 12 cases and out of these, in 6 cases, there was misdiagnosis (in 4 cases, CE was misdiagnosed as AE, and in 2 cases, AE was misdiagnosed as CE). In these studies, the authors claim that an immunohisto/cytochemistry examination with mAb Em2G11 is a relatively successful method in the diagnosis of AE [2]. On the other hand, it is stated that the use of monoclonal antibodies against EM10 in tissue sections is beneficial for distinguishing *E. vogeli*, *E. multilocularis,* and *E. granulosus* [23]. In our cases, which were obtained completely from liver lesions, we have not experienced any histopathological diagnostic difficulties. However, molecular methods along with strain differentiation provide clearer species determination as compared to conventional histopathological examination [22]. PCR on fresh or formalin-fixed, paraffin-embedded tissues is of high interest to confirm the diagnosis of Echinococcosis: *E. multilocularis* or species leading to CE (*E. granulosus* s.s., *E. ortleppi*) [22].

Ultrasound-guided core-needle biopsy has been safely performed at our department for diagnosing hepatic AE for many years. In this study population, the diagnosis could be made without the need for repeat biopsy sampling in any patients. In all seven operable patients, the pathological examinations of the biopsy samples and postoperative pathology results were quite similar. No complications other than short-lived pain were observed in all patients. Needle aspiration in *E. granulosus* cysts is a controversial issue owing to the risks of allergic reactions and anaphylaxis [21]. However, we have not found any data regarding the risk of allergic reactions with percutaneous biopsies from AE lesions. Although we have also not encountered any such reaction in our own studied population, the possibility of allergic reactions in lesions suspicious for AE should be taken into consideration and appropriate medical preparations should be made. Hepatic AE lesions are generally nonvascular, and their percutaneous biopsy is technically simple, since they reach a large size by the time they are detected. We observed no post-procedural bleeding in any of our patients. This can be explained by the characteristics of the lesions mentioned above. Another source of concern is the possibility of seeding living parasitic tissues along the needle tract or causing distal metastatic formations during the procedure [21]. We have not noted any signs of seeding of parasite secondary to biopsy procedures during the visits of our patients for imaging follow-up. However, since there is a risk of recurrence in the following years, follow-up should be continued carefully. Recurrences can reportedly occur after curative operations [7]. Theoretically, percutaneous biopsies can also result in such a consequence. We have not found any literature data regarding this outcome; such a possibility seems very low owing to factors such as weak vascularity of lesions and follow-up of patients under long-term chemotherapy.

There are some limitations of our study. The retrospective nature and the small sample size can be considered the principal limitations. However, AE is a rare disease, and biopsy is performed on only a part of the affected patients. Hence, designing a prospective study enrolling many patients seems difficult. In addition, biopsy procedures were performed by radiologists with different levels of experience. Adequacy assessment, number of entries, puncture site, and biopsy tract were operator-dependent, and, hence, they showed variations. However, these variations had no unfavorable effect on the pathological examination in our study group. Over the years, our pathology department has gained in-depth experience in the diagnosis of both AE and CE. In this study, the tissue specimens derived from our patients were assessed for conventional histopathological features only. The absence of immunohistochemical examination and PCR analysis can be mentioned as another limitation of our study.

Although hepatic AE lesions do have some specific imaging characteristics, they may mimic malignant processes owing to their aggressive radiological appearances. Despite a large liver mass, a relatively good overall clinical condition, normal cancer markers of a patient, and the fact that the patient lives in an endemic region, together support the diagnosis of AE. Histopathological examination can be used to confirm the AE diagnosis in suspected cases that have atypical imaging characteristics and/or other concurrent diseases. Even though ultrasound-guided biopsy gives clear results, it should be done only in selected patients. Although conventional histopathological examination is sufficient to correctly diagnose AE in most cases, recent literature data indicate that in uncertain difficult cases, the histopathological examination should be supported by immunohistochemical examination and PCR. In conclusion, ultrasound-guided core-needle biopsy is a minimally invasive, reliable, and effective diagnostic tool that can be used for the definitive diagnosis of hepatic AE lesions.

## Conflict of interest

The authors declare no conflict of interest in relation with this paper.
